# TeaPVs: a comprehensive genomic variation database for tea plant (*Camellia sinensis*)

**DOI:** 10.1186/s12870-022-03901-5

**Published:** 2022-11-03

**Authors:** Yanlin An, Xiaoqin Zhang, Sixia Jiang, Jingjing Zhao, Feng Zhang

**Affiliations:** Department of Food Science and Engineering, Moutai Institute, Luban Street, Renhuai, 564502 Guizhou People’s Republic of China

**Keywords:** Tea plant, Variations, Resequence, Genome and transcriptome, Database

## Abstract

Genome variation not only plays an important role in plant phenotypic modeling and adaptive evolution, but also enhances population genetic diversity and regulates gene expression. The tea tree (*Camellia sinensis*) has a large genome (~ 3.0 Gb), making the identification of genome-wide variants time-consuming and expensive. With the continuous publication of a large number of different types of population sequencing data, there is a lack of an open platform to integrate these data and identify variants in the tea plant genome.

To integrate the genetic variation confidence in the tea plant population genome, 238 whole-genome resequencing, 213 transcriptome sequencing, and 96 hybrid F1 individuals with a total of more than 20 Tb were collected for mutation site identification. Based on these variations information, we constructed the first tea tree variation web service database TeaPVs (http://47.106.184.91:8025/ and http://liushang.top:8025/). It supports users to search all SNP, Indel, SV mutations and SSR/Polymorphic SSR sequences by location or gene ID. Furthermore, the website also provides the functions of gene expression search of different transcriptome, sequence blast, sequence extraction of CDS and mutation loci, etc.

The features of the TeaPVs database make it a comprehensive tea plant genetic variation bioinformatics platform for researchers, and will also be helpful for revealing new functional mutations in the tea plant genome and molecular marker-assisted breeding.

**Supplementary Information:**

The online version contains supplementary material available at 10.1186/s12870-022-03901-5.

## Background

Tea plant [*Camellia sinensis* (L.) O. Kuntze] is a perennial evergreen woody plant with important economic value originating in southwest China [1]. Tea beverages have become the most popular non-alcoholic beverages in the world due to their rich content of amino acids, catechins and caffeine and other active substances that are beneficial to the human body [2]. According to reports, more than 300 tea varieties have been bred in China, and more than 3000 tea germplasm have been collected and preserved in China National Germplasm Tea Repository. In addition, there are still many wild and ancient tea germplasm to be excavated and identified [3, 4]. Abundant tea plant germplasm also exhibits diverse phenotypic, resistance and quality characteristics. However, despite many research efforts, the formation mechanisms of these important agronomic and quality traits have not been fully resolved.

There are extensive genetic variations in population gene pool, and many studies have shown that these mutations may cause different phenotypes, resistance and quality characteristics of plants. Among them, the two most abundant mutation types on the genome are SNP and Indel. In the early studies, the genotype verification of different varieties and functional gene mapping were mainly carried out through the development of molecular markers including SSR、AFLP and RAPD etc. [5]. However, the SRR marker, as one of the most common genetic markers, can also be regarded as a special Indel marker [6, 7]. In recent years, due to the advancement of sequencing technology and the further reduction of sequencing costs, the discovery of mutations and functional studies based on population resequencing has received increasing attention. For example, Lu et al. resequenced 588 *Brassica napus* and identified 5,294,158 SNPs and 1,307,151 Indels [8]; Cheng et al. reported that a nonsynonymous single nucleotide mutation in *GID1c* disrupted its interaction with *DELLA1*, resulting in a GA-insensitive dwarf phenotype in peach [9]. In addition, the structural variation of large segments is also considered to have an important impact on plant characters. As found in maize, the expression level of Zm00015a037064 may be regulated by a 1794 bp SV [10]; while a 1.67 Mb inversion downstream of a *PpOFP1* gene can lead to changes in peach fruit shape [11, 12].

The progress of sequencing technology has also strongly promoted the research of plant genomes and pan-genomes. Up to now, more than 140 plant genomes have been published (https://www.plabipd.de/index.ep). Since the release of the first tea tree draft genome “Yunkang 10” in 2017 [1], the genomes of seven tea tree varieties, including “Shuchazao” [13]、“Longjing 43” [14]、“Biyun” [15]、“Tieguanyin” [16]、“Huangdan” [17] and “DASZ” [18] have been published successively, providing a basis for whole-genome resequencing research. In 2019, liu et al. identified 7,511,731 SNPs and 255,218 Indels mutations in “Yunkang 10” for the first time by whole-genome resequencing, and developed 48 polymorphic Indel markers [19]. Since then, a large number of tea tree population resequencing and transcriptome data have been published, which has enhanced people’s understanding of the formation mechanism of tea tree quality characteristics and evolutionary history. However, how to make ordinary researchers effectively mine these massive data still faces many difficulties.

For tea trees, some databases have been successfully constructed in previous studies. For example, Xia et al. constructed the first tea tree genome database [20]; Zhang et al. collected 261 high-quality RNA-Seq experiments to construct a tea plant gene co-expression database [21]; Mi et al. collected 66 different tea tree transcriptome datasets to construct a rich alternative splicing database [22]; and Singh et al. constructed the first-generation tea plant haplotype map website [23]. However, compared with other crops [24, 25], the genome-wide variation database of tea plant population remains unreported. In this study, we collected 238 tea plant whole genome resequencing data, 213 transcriptome sequencing data, and 96 hybrid F1 generation resequencing to construct a comprehensive tea plant population variation database. In addition, this database also includes SSR data for six tea tree genomes and SV data for five genomes identified based on Pacbio sequencing data. The successful construction of this database will provide strong support for tea tree genetics and breeding、QTL mapping and functional verification of mutation loci.

## Construction and content

### Data sources

In order to construct a relatively complete database, we collected the genome assembly and Pacbio data of 6 tea plant varieties, including “Shuchazao” [14], “Longjing43” [14], “Tieguanyin” [16], “Biyun” [15], “Huangdan” [17], “DASZ” [18] and “Yunkang 10” [1]; 238 whole-genome resequencing datasets (Additional file 1: Table S1) [4, 13, 16]; a F1 hybrid population with 96 offspring [2]; 213 transcriptome sequencing data (Additional file 2: Table S2) [18]. The above data were used for the identification of SNPs, Indels, SSRs and SVs; an additional 66 transcriptomes were collected from NCBI (https://www.ncbi.nlm.nih.gov/) for expression abundance calculations (Additional file 3: Table S3) [22].

### SNP and Indel variations identification and annotation

For the whole genome resequencing data, refer to the study of Xia et al. [13, 25] to identify the diversity variants, and then use GATK to filter the original variations data set with the following parameters: --minDP 5 --maxDP 100 --minGQ 10 --minQ 30 --min-meanDP 7；The transcriptome data is aligned with the genome twice using STAR software, and after removing the duplicated sequence of the bam file, the HaplotypeCaller module and the CombineGVCFs module in the gatk package are used to generate gvcf files and raw variant datasets, and finally use -minDP 5 -- maxDP 100 --minGQ 10 --minQ 30 --min-meanDP 4 parameters to filter to obtain the final variant dataset. All variants above are annotated using ANNOVAR [26] with default parameters.

### Identification of SVs and SSR sites

Pacbio sequencing data generated by previous genome project was aligned to the reference genome of “Shuchazao” by Minimap2 [27], and samtools [28] was used to convert sam files into bam files and sort bam files. Finally, the default parameters of cuteSV are used to identify the whole genome SV variations [29]. The MISA software (https://webblast.ipk-gatersleben.de/misa/) is used for the identification of genomic SSRs. In order to ensure the accuracy of the identification results, the repeating times of the dinucleotide repeating unit are not less than 6 times, and the trinucleotide, tetranucleotide, pentanucleotide and six Nucleotide repeat unit repeats no less than 5 times. In addition, we used the SSRMMD software to predict the polymorphic SSRs present between the two genomes with default parameters [30].

### Calculation of transcriptome expression levels for different treatments

First, the script provided by hisat2 is used to extract splicing site and exon information. After establishing genome index by hisat2, the filtered clean reads are aligned to the reference genome, and the sam files are converted into bam format and sorted. Assemble the transcript with the default parameters of stringtie, and extract TPM from the result file to represent the gene expression level [31].

### Database construction

In order to build interactive web services quickly, we use a brand-new micro web framework based on Flask: Streamlit (https://streamlit.io/)，which is widely used for machine learning and data sharing. Different from the web construction mode with front-end separation, it can not only refer to bootstrap and html to complete the design of web pages, but also provide all interactive functions. This provides a foundation for the rapid completion of database development. The aggrid plugin provides beautification and additional query functionality for tables. In addition, pandas completed the query function of the server, while the extraction and alignment of sequences are completed by seqtk and blast respectively. All raw data is stored on Ubuntu 20.04 LTS server system.

## Utility and discussion

### Database overview

**Fig. 1:**
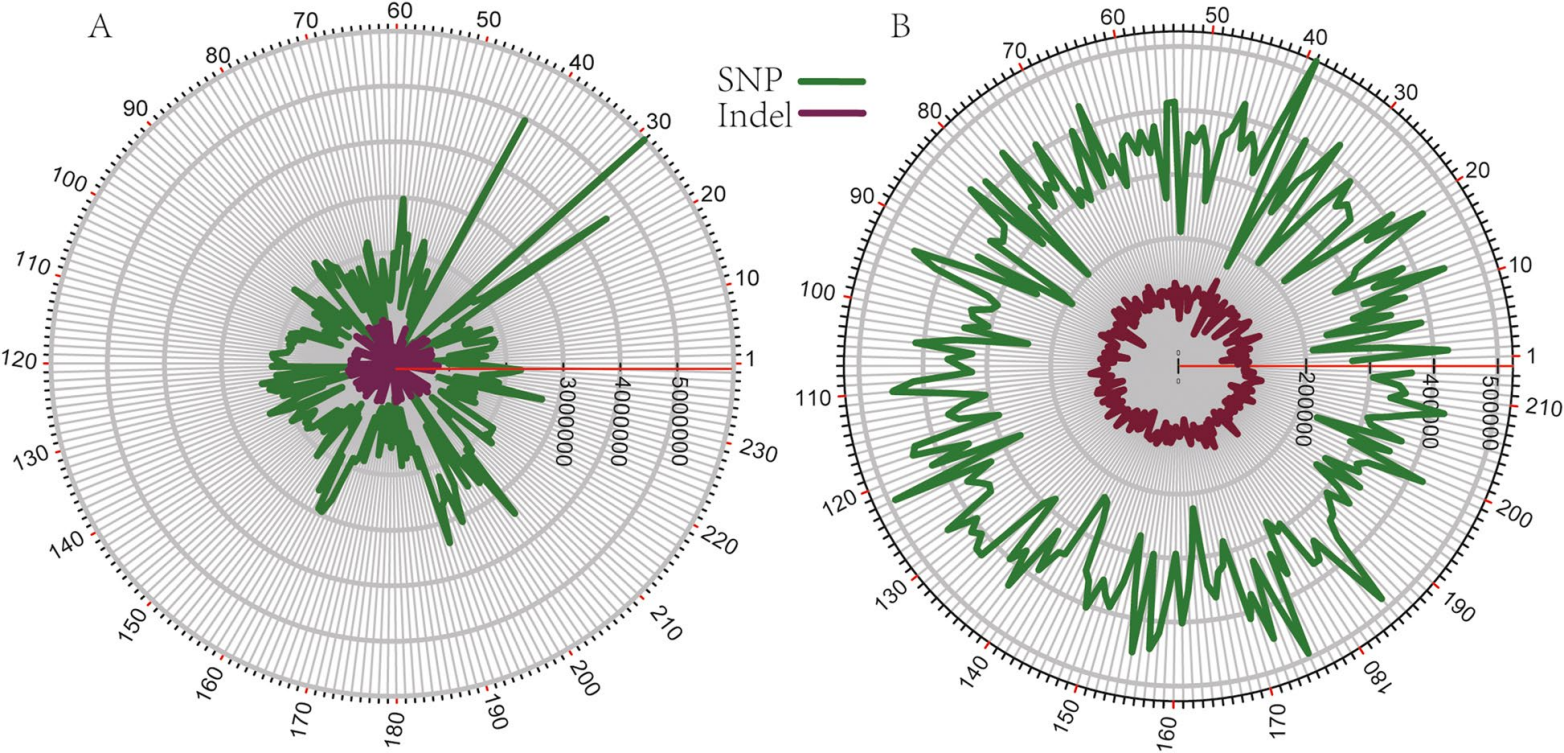
The number of SNPs and Indels in resequencing and transcriptome sequencing samples, and the sample names corresponding to the numbers can be viewed in Table. S1, Table. S2. Figure A and B represent resequencing and transcriptome sequencing, respectively

To construct this database, more than 20 Tb of sequencing readings and 6 tea plant genomes were collected and reanalyzed. In 238 whole-genome resequencing datasets, 11,469,723 SNPs and 4,997,785 Indels were identified, and each sample contained SNPs and Indels ranging from 41,443 to 5,986,103 and 3944 to 810,753, respectively (Fig. 1A). The difference of sequencing depth results in great differences of mutation sites among samples. In the 213 transcriptome datasets, 6,757,348 SNPs and 2,072,762 Indels were identified, and each sample contained SNPs and Indels ranging from 173,753 to 523,577 and 137,963 to 307,062, respectively (Fig. 1B); In the 96 hybrid F1 resequencing populations, a total of 1,022,684 SNPs were identified, of which 623,054 and 546,276 SNPs were identified in the maternal and paternal parents, respectively, and the average SNP content of the progeny was 404,704 (Additional file4: Fig. S1A). At the same time, six tea plant genomes with SSR content at 441,549 to 595,418 (Additional file 4: Fig. S1B), while the SV numbers of five tea plants ranged from 32,094 to 351,124 (Additional file 4: Fig. S1C). The reason for the large difference in the number of structural variations is not only related to the sample itself, but also closely related to the amount of sequencing data. In addition, 63 sets of transcriptomes with different treatments were aligned to the “Shuchazao” reference genome, and the expression levels of all genes in the genome under different treatments were calculated based on the aligned bam files.

### Web overview

**Fig. 2:**
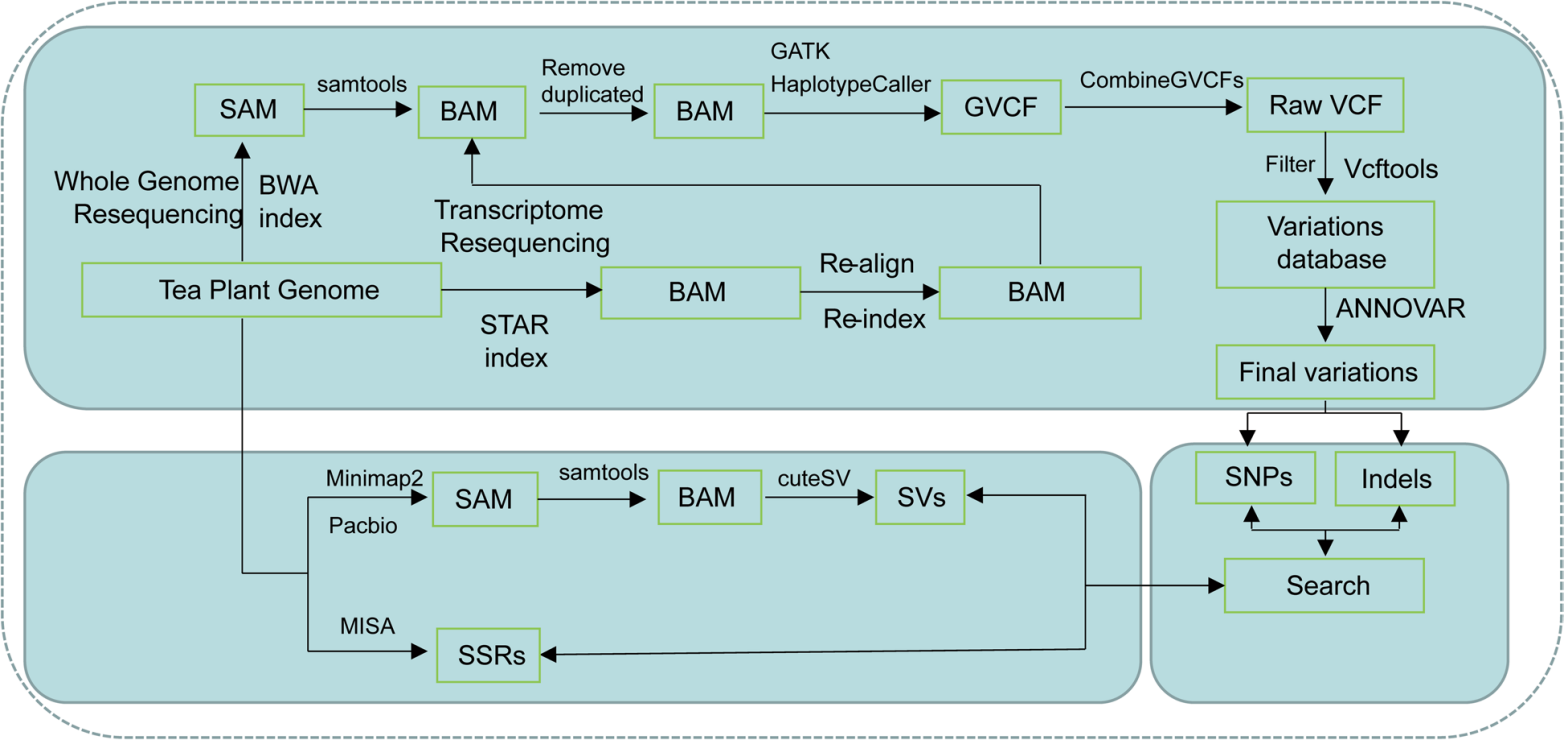
Workflow of the construction of the TeaPVs database

Multiple steps are taken and implemented to build a sufficiently robust web service database. The TeaPVs database integrates natural and hybrid population whole-genome resequencing, population transcriptome resequencing, transcriptome expression, and genome data to provide a highly available tea plant variation database. The specific integration steps are shown in Fig. 2. The TeaPVs website provides two main sections including Search module and Tools module located in sidebar region. In the search module, four sub-function options are provided: SNPs/Indels search、SVs search、Polymorphic SSRs search and Transcription abundance search; While the Tools module provides Blast、Extract sequences and Download functions. Panels outside the Sidebar area are used as the view area to return the corresponding results when performing search or tools functions, otherwise the introduction information of TeaPVs web is displayed.

### Usage cases

**Fig. 3:**
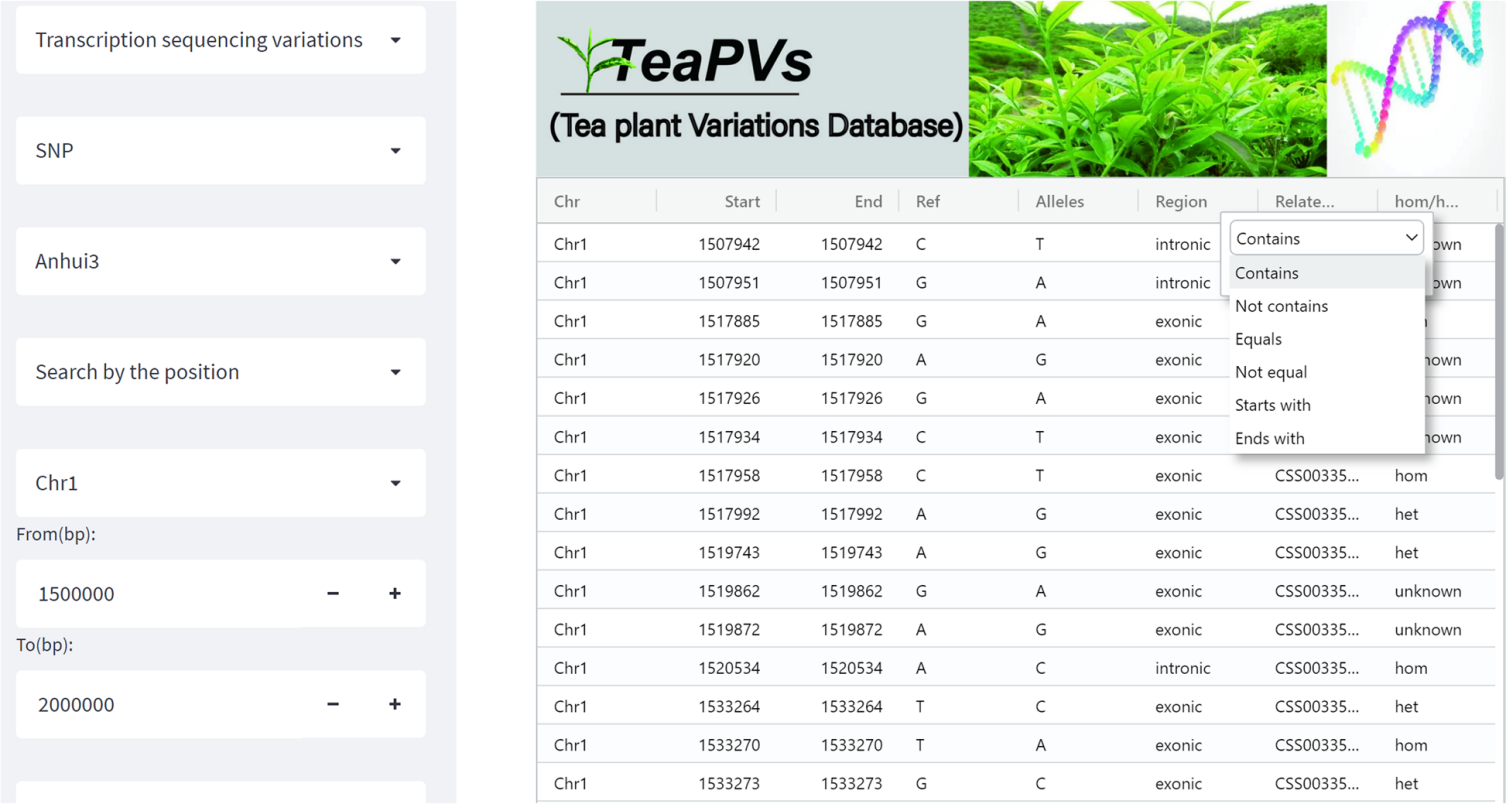
Search tools and examples of results

Case study 1, in the sidebar SNP/Indel search sub-search module, users can search for SNP/Indel by selecting any one of the three data sources “Transcription sequencing variations”, “Re-sequencing variations” and “F1 sequencing variations”. When the data source is selected, determine the type of variant to search (SNP or Indel), and then select a sample name. Next, if the user chooses to search by position, they need to select the chromosome number and enter the interval information. As shown in Fig. 3, the SNP mutation of transcriptome data source was selected to search, and the interval of 1500,000 bp to 200,000 bp of chromosome 1 of “Anhui 3” sample was selected, and then the search results were displayed in the right view area; Otherwise, the user needs to enter a gene ID, for example, the Go annotation results show that the CSS0001553 (The gene ID is defined in the “Shuchazao” reference genome [13], http://tpia.teaplant.org/) gene may respond to plant cold stress, and when the correct gene ID is entered, one synonymous mutation and two non-synonymous mutations are displayed. In particular, each column of the result table has a search function. When there are many mutation sites in the interval, it can be further filtered according to the genotype of the “Ref” column and the “Alleles” column or the “Region” and “Effect” obtained by the annotation.

**Fig. 4:**
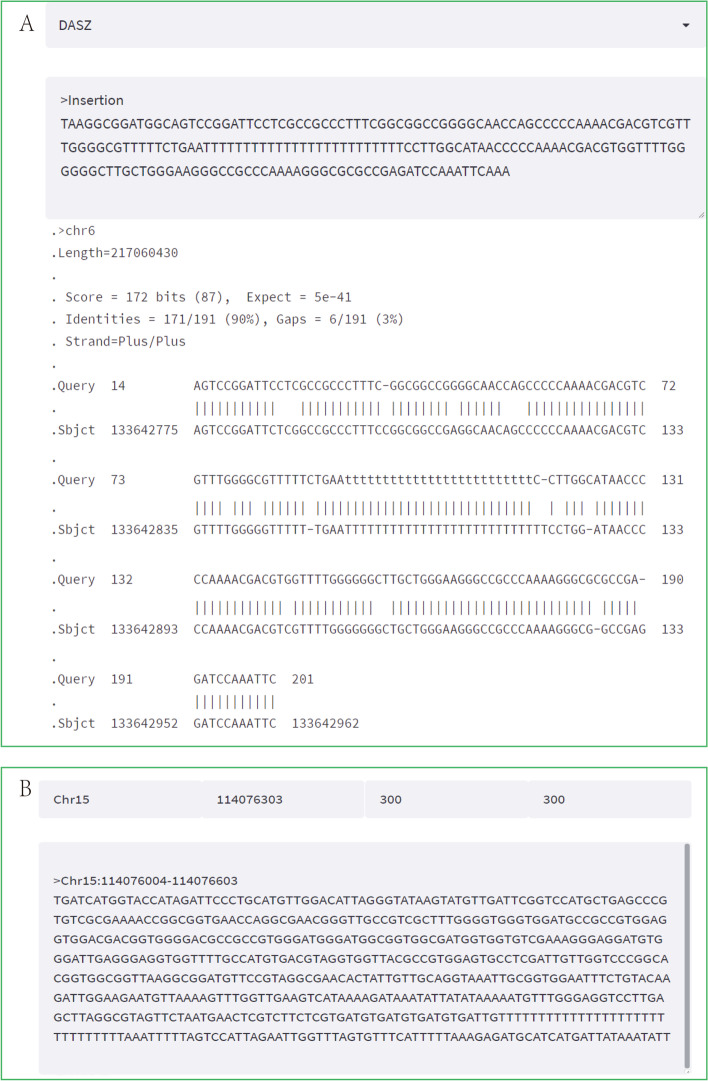
Examples of blast and sequence extraction tools

Case study 2, Blast, as a common sequence alignment program, is also provided in the Tools module. In the SVs sub-search module, after selecting the “Longjing 43” genome, the structural variation between the tea variety and the reference genome can be searched. For example, at 114,076,303 bp of chromosome 15, an insertion with a length of 203 bp located in the exon region of CSS0026339 was identified. Users can select any one of the six tea tree genomes as database, and then enter the insertion sequence in fasta format, and the alignment results will be displayed in the view interface (Fig. 4A). If further verification is needed, in the “extracting sequences” submodule, the target sequence can be obtained by entering the specific position of the mutation locus and the two position parameters before and after it (Fig. 4B). Then, suitable primers can be designed online using Primer3 (https://bioinfo.ut.ee/primer3-0.4.0/).

In addition, we also provide SSR sequence information of six tea plant genomes, especially users can search for polymorphic SSR by selecting different genome pairs. When a mutation or sequence is considered to be related to gene expression level, users can view the expression level of the corresponding gene in different transcriptome experiments through“Transcript abundance search”. At the same time, the “Extrac CDS” and “Download” functions allow users to extract CDS sequences of different genes and to download all SNP, Indel, SV, SSR and expression level files contained in this database.

## Conclusions

In recent years, tea plant multi-omics sequencing data have been published [32, 33], but the integration and comprehensive utilization of these data is more difficult than other species due to the tea plant genome size of about 3 Gb and high heterozygosity [34, 35]. In this study, we integrated more than 20 Tb of sequencing data from multiple data sources to build a powerful database of tea plant variation. The successful release of TeaPVs database will provide strong support for molecular marker-assisted breeding and gene function research of tea tree. At the same time, it is also a continuously updated project. With more data being analyzed and the latest sequencing data being published continuously, more variation information will be added to the database for all users to search.

## Supplementary Information

**Additional file 1:** **Table. S1.** Resequencing samples and their corresponding numbers.

**Additional file 2:** **Table. S2.** Transcriptome sequencing samples and their corresponding numbers.

**Additional file 3:** **Table. S3.** Different treatment transcriptome sample names.

**Additional file 4:** **Fig. S1.** Statistics of SNPs, genomic SSRs and SVs in the F1 population. Fig. S1A represents the number of SNPs identified in each sample of the F1 population; Fig. S1B and Fig. S1C represent the number of SSRs and SVs identified in the corresponding genome, respectively.

## Data Availability

The genomic data of the tea plant (*Camellia sinensis*) are available at Tea Plant Information Archive (TPIA, http://tpia.teaplant.org/download.html) and Tea Plant Genome Database (TeaPGDB, http://eplant.njau.edu.cn/tea). The re-sequencing (PRJNA716079, PRJNA597714, PRJNA665594)、F1 generation sequencing (PRJNA727668) and RNA-seq datasets (PRJNA595795) supporting the results of this article are available at the SRA database of National Center for Biotechnology Information (NCBI, https://www.ncbi.nlm.nih.gov/). The database code can be obtained from https://gitee.com/qiushui1234567/database-code.

## Abbreviations

TeaPVs: Tea plant variations database
SNP: Single nucleotide polymorphism
Indel: Insertion-deletion
SV: Structure Variantion
TPM: Transcripts per million reads
CDS: Coding sequence
