# Supplementary figures and images for: TeaPVs: a comprehensive genomic variation database for tea plant (Camellia sinensis)
Source: BMC Plant Biol. 2022 Nov 3;22:513. doi: 10.1186/s12870-022-03901-5 (PMC9632082; doi:10.1186/s12870-022-03901-5)

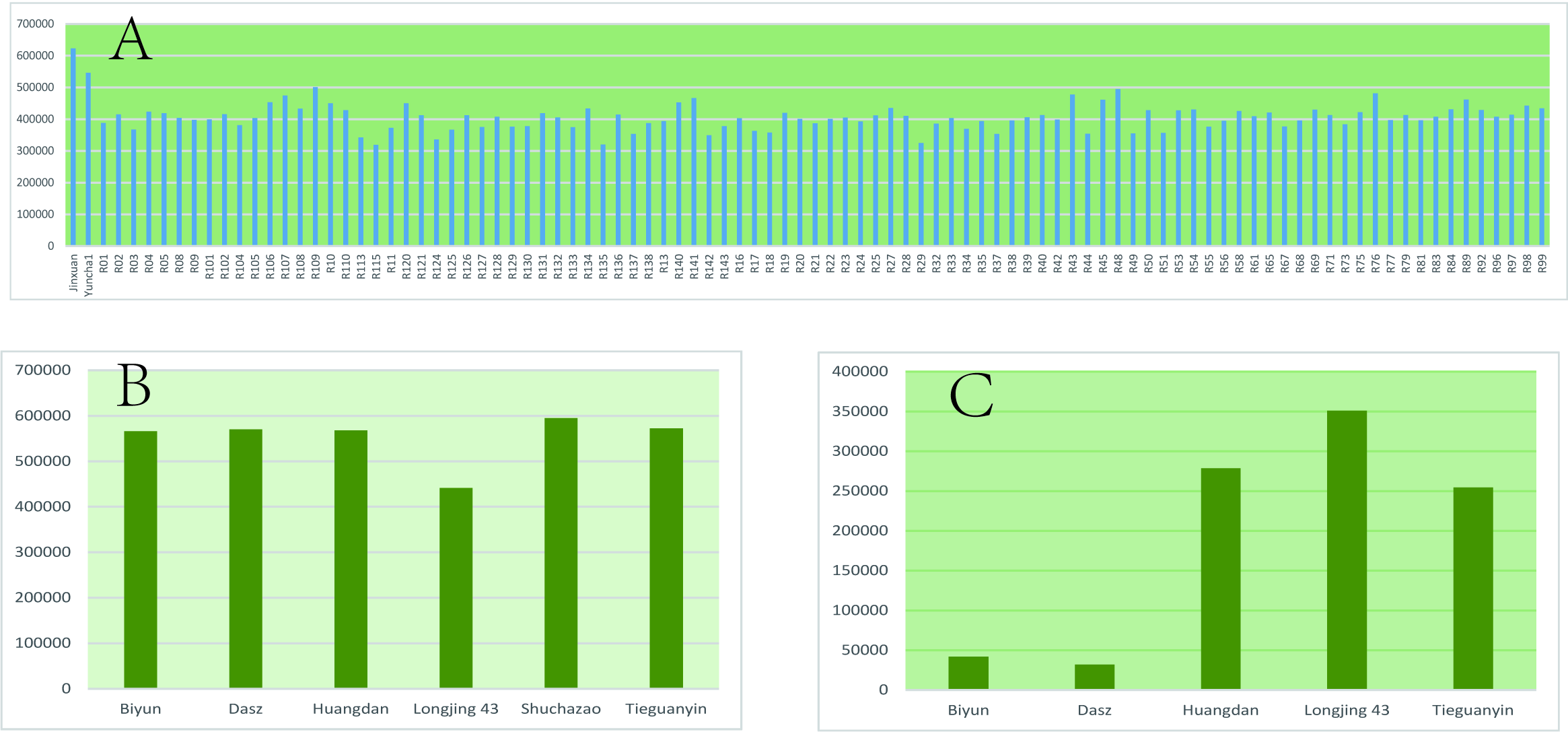

Supplement: Supplementary file 4 — Additional file 4: Fig. S1. Statistics of SNPs, genomic SSRs and SVs in the F1 population. Fig. S1A represents the number of SNPs identified in each sample of the F1 population; Fig. S1B and Fig. S1C represent the number of SSRs and SVs identified in the corresponding genome, respectively. [file 12870_2022_3901_MOESM4_ESM.tif]
